# Device Performance of a Tubular Membrane Dialyzer Incorporating Ultrafiltration Effects on the Dialysis Efficiency

**DOI:** 10.3390/membranes13060556

**Published:** 2023-05-28

**Authors:** Chii-Dong Ho, Jr-Wei Tu, Jun-Wei Lim, Wei-Chi Lai

**Affiliations:** 1 Department of Chemical and Materials Engineering, Tamkang University, Tamsui, New Taipei 251301, Taiwan; 891360033@gms.tku.edu.tw (J.-W.T.); wclai@mail.tku.edu.tw (W.-C.L.); 2 Department of Fundamental and Applied Sciences, HICoE-Centre for Biofuel and Biochemical Research, Institute of Self-Sustainable Building, Universiti Teknologi PETRONAS, Seri Iskandar 32610, Perak Darul Ridzuan, Malaysia; junwei.lim@utp.edu.my

**Keywords:** dialysis, tubular dialyzer, ultrafiltration, mass transfer, separation efficiency

## Abstract

Membrane dialysis is one of the membrane contactors applied to wastewater treatment. The dialysis rate of a traditional dialyzer module is restricted because the solutes transport through the membrane only by diffusion, in which the mass-transfer driving force across the membrane is the concentration gradient between the retentate and dialysate phases. A two-dimensional mathematical model of the concentric tubular dialysis-and-ultrafiltration module was developed theoretically in this study. The simulated results show that the dialysis rate improvement was significantly improved through implementing the ultrafiltration effect by introducing a trans-membrane pressure during the membrane dialysis process. The velocity profiles of the retentate and dialysate phases in the dialysis-and-ultrafiltration system were derived and expressed in terms of the stream function, which was solved numerically by the Crank–Nicolson method. A maximum dialysis rate improvement of up to twice that of the pure dialysis system ($V_{w}=0$) was obtained by employing a dialysis system with an ultrafiltration rate of $V_{w}=2\;mL/min$ and a constant membrane sieving coefficient of θ=1. The influences of the concentric tubular radius, ultrafiltration fluxes and membrane sieve factor on the outlet retentate concentration and mass transfer rate are also illustrated.

## 1. Introduction

Membrane extraction [1,2], membrane absorption [3,4], ion-exchange membranes [5,6] and membrane distillation [7,8] are membrane-based separation processes [9] which have been widely applied to many industries over decades in concentration or purification processes, in which the important features of low energy consumption, continuous operation and easy up-scaling [10,11] were the main advantages of these membrane technologies. However, membrane dialysis is unique among the membrane contactors in that the solvent may transport through the membrane accompanied by the solutes from the feed stream (defined as the retentate phase) to the receiving stream (defined as the dialysate phase) by diffusion due to the driving force of the concentration gradient. An early use of membrane dialysis was Donnan dialysis [12] and alcohol reduction in beverages [13], and the most famous applications of hemodialysis (artificial kidney) are conducted to remove waste metabolic end products from the human body [14,15]. Hemodialysis is a process of purifying blood through hemodialyzers containing a hemodialysis membrane, which is the most effective treatment to prolong the life of people suffering from end-stage renal diseases (ESRD) [16].

The dialysate phase concentration in a dialysis process does not remain unchanged and the solvent is inevitably to pass through the membrane, across which exists a pressure gradient between two phases besides the other membrane contactors. Early studies focused on a simplified analysis by ignoring the concentration variance of the dialysate phase and assuming no solvent passed through the membrane [17,18]. A two-dimensional theoretical model was developed [19] to simulate the mass transfer in hemodialyzers considering concentration variations and the overall mass-transfer coefficients were determined in an unsteady-state recycle dialyzer by neglecting the concentration change [20]. The effect of the ultrafiltration operation on the mass-transfer efficiency improvement was studied by simultaneously diffusing and convecting the solute in a dialyzer while assuming there was no solute concentration in the dialysate phase [21] and nonzero dialysate concentration [22]. Henderson [23] and Leber et al. [24] have shown that the ultrafiltration operation not only can improve the mass transfer efficiency in a hemodialysis process but also can reduce the treatment time; in addition, it is tolerated by patients. Furthermore, Yeh et al. [25] investigated the mass transfer in a cross-flow parallel-plate dialyzer coupled with ultrafiltration by using the perturbation method. A one-dimensional theoretical model of a concurrent-flow [26] and countercurrent-flow [27] parallel-plate dialyzer coupled with ultrafiltration was developed and solved by using the Frobenius method. Successful intensifications of membrane dialyzers have been developed and employed in anion-exchange membranes for acid recovery from acidic wastewater [28] and heparin-free hemodialysis [29,30]. Previous studies proved some functionalized hemodialysis membranes were used to overwhelm oxidative stress [31] hemodialysis therapy, in which utilizing a tannic acid (TA) coating as a reactive oxygen species was employed in membrane dialysis indicating an increase in the serum total antioxidant capacity. Moreover, the anion-exchange membranes for acid recovery depend on the dialysis efficiency and selectivity of membrane gas absorption, distribution coefficient and a composition gradient of gas solute in the gas/liquid system [32].

The phenomena of suction or injection through the membrane due to the ultrafiltration operation will lead to a complex flowing pattern in the channels of a membrane dialyzer, in which the velocity distribution can be solved using the stream function coupled with the perturbation method [33,34]. A two-dimensional mathematical model of the concentric tubular dialysis-and-ultrafiltration module is developed theoretically. Though the mass-transfer characteristics in the present study could be analogized from those in our previous work on the flat-plate module [35], the manners of velocity and transport across the membrane are somehow different and the mass-transfer mathematical formulation is relatively complicated. This study actually extends the one-dimensional modeling of the flat-plate module to a two-dimensional analysis of the concentric tubular dialysis-and-ultrafiltration system in the present study to obtain improvements in dialysis efficiency and rate; however, no theoretical study in its simplified expression has been reported in the literature for a concentric tubular dialyzer with an ultrafiltration operation. The general applicability of such a mathematical formulation was investigated in a manner analogous to the parallel-plate dialysis-and-ultrafiltration system [26] to analyze the concentric tubular dialyzer module in this study. The purpose of the present study is to discuss the effects of the retentate phase flow rate, dialysate phase flow rate, ultrafiltration rate, membrane sieving coefficient and channel thickness ratio on the dialysis efficiency in a concentric tubular dialysis-and-ultrafiltration system. This is the value of the present study to delineate a two-dimensional concentric tubular dialyzer under ultrafiltration operation with such a simplified mathematical formulation.

## 2. Mathematical Formulations

### 2.1. Dialysis-and-Ultrafiltration Operation in a Concentric Tubular Module

**Figure 1:**
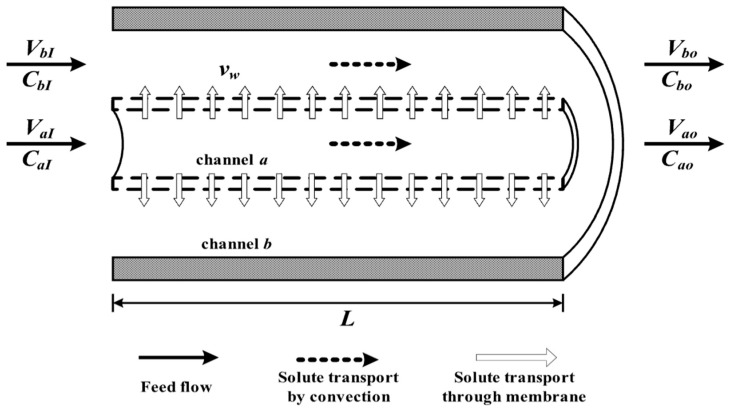
Concentric tubular dialysis-and-ultrafiltration module.

A hydrophilic membrane with thickness δ was inserted in parallel as the inner tube into a circular tube with length L to divide the open conduit into parts: an inner subchannel *a* (retentate phase) and annular subchannel *b* (dialysate phase) with inner radius $r_{t}$ and $r_{s}$, respectively, as shown in Figure 1 which indicates the solutes transporting through the membrane by diffusion for mass transfer. An ultrafiltration flow with flow rate $V_{w}$ and with inlet flow rates $V_{aI}$ and $V_{bI}$ of the retentate and dialysate phases, respectively, was introduced in the concentric tubular dialyzer instead of omitting the ultrafiltration operation, leading to a dialysis rate improvement with channel thickness ratio and membrane sieving coefficient as parameters, as shown in Figure 1. The theoretical analysis is based on the following assumptions: (a) constant physical properties of fluid; (b) a fully developed laminar flow on the entire length in the inner and annular subchannels; (c) negligible entrance length and end effects; (d) negligible longitudinal diffusion; (e) constant membrane sieving coefficient θ.

#### 2.1.1. Velocity Distributions

The velocity distributions were derived with the use of the continuity equations and momentum balance equations in both inner subchannel *a* and annular subchannel *b* under the assumed conditions as follows:

(1) $$\frac{u_{ar}}{r}+\frac{\partial u_{ar}}{\partial r}+\frac{\partial u_{az}}{\partial z}=0$$

(2) $$\frac{u_{br}}{r}+\frac{\partial u_{br}}{\partial r}+\frac{\partial u_{bz}}{\partial z}=0$$

(3) $$u_{az}\frac{\partial u_{az}}{\partial z}+u_{ar}\frac{\partial u_{az}}{\partial r}=-\frac{1}{\rho }\frac{\partial p_{a}}{\partial z}+\upsilon \left(\frac{\partial^{2}u_{az}}{\partial r^{2}}+\frac{\partial u_{az}}{r\partial r}+\frac{\partial^{2}u_{az}}{\partial z^{2}}\right)$$

(4) $$u_{az}\frac{\partial u_{ar}}{\partial z}+u_{ar}\frac{\partial u_{ar}}{\partial r}=-\frac{1}{\rho }\frac{\partial p_{a}}{\partial r}+\upsilon \left(\frac{\partial^{2}u_{ar}}{\partial r^{2}}+\frac{\partial u_{ar}}{\partial r}-\frac{u_{ar}}{r^{2}}+\frac{\partial^{2}u_{ar}}{\partial z^{2}}\right)$$

(5) $$u_{bz}\frac{\partial u_{bz}}{\partial z}+u_{br}\frac{\partial u_{bz}}{\partial r}=-\frac{1}{\rho }\frac{\partial p_{b}}{\partial z}+\upsilon \left(\frac{\partial^{2}u_{bz}}{\partial r^{2}}+\frac{\partial u_{bz}}{r\partial r}+\frac{\partial^{2}u_{bz}}{\partial z^{2}}\right)$$

(6) $$u_{bz}\frac{\partial u_{br}}{\partial z}+u_{br}\frac{\partial u_{br}}{\partial r}=-\frac{1}{\rho }\frac{\partial p_{b}}{\partial r}+\upsilon \left(\frac{\partial^{2}u_{br}}{\partial r^{2}}+\frac{\partial u_{br}}{\partial r}-\frac{u_{br}}{r^{2}}+\frac{\partial^{2}u_{br}}{\partial z^{2}}\right)$$

The dimensionless forms of the velocity distributions, $u_{a\xi }$ and $u_{a\eta }$, are the velocity profiles in the longitudinal and perpendicular directions in the inner subchannel incorporating with the dimensionless groups, $∆=\frac{r_{t}}{r_{s}},\eta =\frac{r}{r_{s}}$ and $\xi =\frac{z}{L}$, $\bar{u_{aI}}=\frac{V_{aI}}{\pi r_{t}^{2}},v_{w0}=\frac{V_{w}}{2\pi r_{t}L},\lambda_{a}=\frac{\Delta r_{s}v_{w0}}{\upsilon }$, which were derived in Appendix A as follows:

(7) $$u_{a\xi }=[2\Delta^{2}\bar{u_{aI}}-4\frac{\Delta v_{w0}L}{r_{s}}\xi ]\left\{\frac{\left(\Delta^{2}-\eta^{2}\right)}{\Delta^{4}}\right.+\lambda_{a}\left[\frac{1}{18\Delta^{2}}-\frac{\eta^{2}}{4\Delta^{4}}+\frac{\eta^{4}}{4\Delta^{6}}-\frac{\eta^{6}}{18\Delta^{8}}\right]\;\left.+\lambda_{a}^{2}\left[\frac{83}{5400\Delta^{2}}-\frac{19(\eta^{2})}{270\Delta^{4}}+\frac{11(\eta^{4})}{144\Delta^{6}}-\frac{\eta^{6}}{36\Delta^{8}}+\frac{\eta^{8}}{144\Delta^{10}}-\frac{\eta^{10}}{1800\Delta^{12}}\right]\right\}$$

(8) $$u_{a\eta }=2\Delta v_{w0}\left\{\frac{1}{\Delta^{4}}\left(\Delta^{2}\eta -\frac{\eta^{3}}{2}\right)+\lambda_{a}\left[\frac{\eta }{18\Delta^{2}}-\frac{\eta^{3}}{8\Delta^{4}}+\frac{\eta^{5}}{12\Delta^{6}}-\frac{\eta^{7}}{72\Delta^{8}}\right]\right.\;+\lambda_{a}^{2}\left[\frac{83\eta }{5400\Delta^{2}}-\frac{{19\eta }^{3}}{540\Delta^{4}}+\frac{{11\eta }^{5}}{432\Delta^{6}}-\frac{\eta^{7}}{144\Delta^{8}}++\frac{\eta^{9}}{720\Delta^{10}}-\frac{\eta^{11}}{10,800\Delta^{12}}\right]$$

Similarly, the dimensionless forms of velocity distributions in the annulus subchannel, $u_{b\xi }$ and $u_{b\eta }$, were obtained using the same procedure except for the stream function, and were derived in Appendix B with ${∆}_{1}=\frac{r_{o}}{r_{s}},\bar{u_{bI}}=\frac{V_{bI}}{\pi \left(r_{s}^{2}-r_{o}^{2}\right)},v_{w1}=\frac{V_{w}}{2\pi r_{o}L},\lambda_{b}=\frac{\Delta_{1}r_{s}v_{w1}}{\upsilon }$, as follows:

(9) $$u_{b\xi }=\left[2\left(1-\Delta_{1}^{2}\right)\cdot \bar{u_{bI}}+4\frac{\Delta_{1}v_{w1}L}{r_{s}}\xi \right]\left(f_{b0}^{\prime }(\eta )+\lambda_{b}f_{b1}^{\prime }(\eta )\right)$$

(10) $$u_{b\eta }=\frac{2\Delta_{1}v_{w1}}{\eta }\left(f_{b0}(\eta )+\lambda_{b}f_{b1}(\eta )\right)$$

#### 2.1.2. Mass Balance Equations

**Figure 2:**
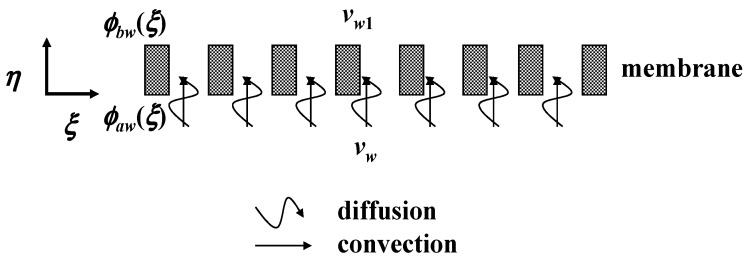
Concentric tubular dialyzer module with ultrafiltration operation.

The mass transfer equations in the retentate, dialysate and membrane phases were derived in the concentric tubular dialyzer with the ultrafiltration system, as shown in Figure 2

(11) $$\frac{u_{a\xi }}{L}\frac{\partial \phi_{a}}{\partial \xi }+\frac{u_{a\eta }}{r_{s}}\frac{\partial \phi_{a}}{\partial \eta }=\frac{D}{r_{s}^{2}}\left(\frac{\partial^{2}\phi_{a}}{\partial \eta^{2}}+\frac{1}{\eta }\frac{\partial \phi_{a}}{\partial \eta }\right)$$

(12) $$\frac{u_{b\xi }}{L}\frac{\partial \phi_{b}}{\partial \xi }+\frac{u_{b\eta }}{r_{s}}\frac{\partial \phi_{b}}{\partial \eta }=\frac{D}{r_{s}^{2}}\left(\frac{\partial^{2}\phi_{b}}{\partial \eta^{2}}+\frac{1}{\eta }\frac{\partial \phi_{b}}{\partial \eta }\right)$$

(13) $$\frac{v_{w}(\eta )}{r_{s}}\frac{\partial \phi_{m}}{\partial \eta }=\frac{D_{m}}{r_{s}^{2}}\left(\frac{\partial^{2}\phi_{m}}{\partial \eta^{2}}+\frac{1}{\eta }\frac{\partial \phi_{m}}{\partial \eta }\right)$$

in which $\phi_{i}=\frac{C_{i}}{C_{aI}}$, and the subscripts *a*, *b* and *m* refer to the retentate, dialysate and membrane phases, respectively; $v_{w}\left(\eta \right)=\frac{1}{\eta }\frac{V_{w}}{2\pi rL}$, $\Delta \leq \eta \leq \Delta_{1}$; δ is the membrane thickness; D is the solute diffusivity in the retentate and dialysate phases; ε is the porosity of the membrane. The corresponding boundary conditions are

(14) $$\phi_{a}(\eta ,0)=1,\;0\leq \eta \leq \Delta$$

(15) $$\frac{\partial \phi_{a}(0,\xi )}{\partial \eta }=0,\;0\leq \xi \leq 1$$

(16) $$\theta \phi_{a}(\Delta ,\xi )=\phi_{m}(\Delta ,\xi ),\;0\leq \xi \leq 1$$

(17) $$-D\frac{\partial \phi_{a}(\Delta ,\xi )}{\partial \eta }+v_{w0}\phi_{aw}(\Delta ,\xi )=-D_{m}\frac{\partial \phi_{m}(\Delta ,\xi )}{\partial \eta }+v_{w0}\phi_{m}(\Delta ,\xi ),\;0\leq \xi \leq 1$$

(18) $$\phi_{b}(\Delta_{1},\xi )=\phi_{m}(\Delta_{1},\xi ),\;0\leq \xi \leq 1$$

(19) $$D\frac{\partial \phi_{b}(\Delta_{1},\xi )}{\partial \eta }=D_{m}\frac{\partial \phi_{m}(\Delta_{1},\xi )}{\partial \eta },\;0\leq \xi \leq 1$$

(20) $$\frac{\partial \phi_{b}(1,\xi )}{\partial \eta }=0,\;0\leq \xi \leq 1$$

(21) $$\phi_{b}(\eta ,0)=\phi_{bI},\;\Delta_{1}\leq \eta \leq 1$$

where θ is the membrane sieving coefficient in which θ=1 means the solute can freely pass through the membrane, and θ means that the solutes are partially rejected by the membrane.

The solute in the membrane phase is simultaneously transported by convection and diffusion due to the dialysis-and-ultrafiltration operation, as shown in Figure 2 and indicated in Equation (13), which can be rewritten as

(22) $$\frac{\partial^{2}\phi_{m}}{\partial t^{2}}+\frac{1}{\eta }\left[1-\frac{V_{w}}{2\pi LD_{m}}\right]\frac{\partial \phi_{m}}{\partial \eta }=\frac{\partial^{2}\phi_{m}}{\partial t^{2}}-\alpha \frac{\partial \phi_{m}}{\partial t}=0$$

in which $\alpha =V_{w}/2\pi LD_{m}$ and t=ln⁡η.

The general solution of Equation (22) is

(23) $$\phi_{m}\left(\eta ,\xi \right)=a_{1}\left(\xi \right)\exp\left(\alpha t\right)+a_{2}\left(\xi \right)=a_{1}{(\xi )\eta }^{\alpha }+a_{2}(\xi )$$

One obtains Equations (24) and (25) for $a_{1}$ and $a_{2}$, respectively, once Equation (23) is substituted into the boundary conditions of Equations (16) and (18):

(24) $$a_{1}(\xi )=\frac{\theta \phi_{aw}(\xi )-\phi_{bw}(\xi )}{\Delta^{\alpha }-\Delta_{1}^{\alpha }}$$

and

(25) $$a_{2}(\xi )=\frac{\Delta^{\alpha }\phi_{bw}(\xi )-\theta \Delta_{1}^{\alpha }\phi_{aw}(\xi )}{\Delta^{\alpha }-\Delta_{1}^{\alpha }}$$

Thus, the solute concentration distribution $\phi_{m}\left(\eta ,\xi \right)$ in the membrane of Equation (23) and the derivative of $\phi_{m}\left(\eta ,\xi \right)$ were obtained as

(26) $$\phi_{m}=\frac{\theta (\eta^{\alpha }-\Delta_{1}^{\alpha })\phi_{aw}(\xi )+(\Delta^{\alpha }-\eta^{\alpha })\phi_{bw}(\xi )}{\Delta^{\alpha }-\Delta_{1}^{\alpha }}$$

(27) $$\frac{\partial \phi_{m}}{\partial \eta }=\alpha \left(\frac{\theta \phi_{aw}(\xi )-\phi_{bw}(\xi )}{\Delta^{\alpha }-\Delta_{1}^{\alpha }}\right)\eta^{\alpha -1}$$

By substituting Equation (27) into the boundary conditions of Equations (17) and (19), the derivatives of $\phi_{a}$ and $\phi_{b}$ were obtained in Equations (28) and (29), respectively:

(28) $$\frac{\partial \phi_{a}}{\partial \eta }=\alpha \varepsilon \left(\frac{\theta \phi_{aw}(\xi )-\phi_{bw}(\xi )}{\Delta^{\alpha }-\Delta_{1}^{\alpha }}\right)\Delta^{\alpha -1}-\left[\frac{r_{s}v_{w0}}{D}\left(\theta -1\right)\right]\phi_{aw}(\xi ),\;0\leq \xi \leq 1$$

(29) $$\frac{\partial \phi_{b}}{\partial \eta }=\alpha \varepsilon \left(\frac{\theta \phi_{aw}(\xi )-\phi_{bw}(\xi )}{\Delta^{\alpha }-\Delta_{1}^{\alpha }}\right)\Delta_{1}^{\alpha -1},\;0\leq \xi \leq 1$$

Hence, the two-dimensional solute concentration distributions of a concentric tubular dialysis-and-ultrafiltration operation can be solved by the governing equations, Equations (11) and (12), with the use of the boundary conditions of Equations (14), (15), (20), (21), (28) and (29).

### 2.2. Pure Membrane Dialysis Operation in a Concentric Tubular Module

**Figure 3:**
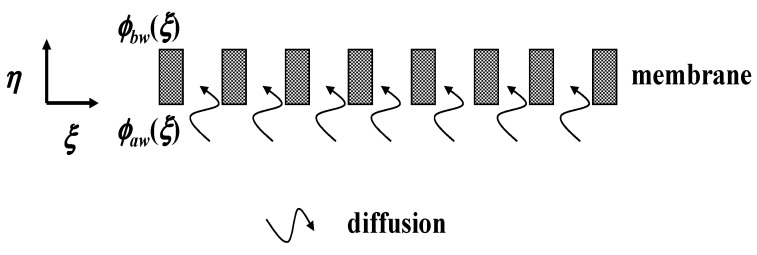
Concentric tubular dialyzer module without ultrafiltration operation.

A concentric tubular dialysis system without ultrafiltration operations, that is, $V_{w}=0$, is a pure membrane dialysis without ultrafiltration operation, as shown in Figure 3. The solute is transported by diffusion only in the membrane phase.

The velocity distributions are the same equations, that is, Equations (7) and (8) and Equations (9) and (10), for the inner subchannel *a* (retentate phase) and annular subchannel *b* (dialysate phase), respectively, except for incorporating $V_{w}=0$. The governing equations of the pure dialysis system in the retentate, dialysate and membrane phases are

(30) $$\frac{u_{a\xi }}{L}\frac{\partial \phi_{a}}{\partial \xi }=\frac{D}{r_{s}^{2}}\left(\frac{\partial^{2}\phi_{a}}{\partial \eta^{2}}+\frac{1}{\eta }\frac{\partial \phi_{a}}{\partial \eta }\right)$$

(31) $$\frac{u_{b\xi }}{L}\frac{\partial \phi_{b}}{\partial \xi }=\frac{D}{r_{s}^{2}}\left(\frac{\partial^{2}\phi_{b}}{\partial \eta^{2}}+\frac{1}{\eta }\frac{\partial \phi_{b}}{\partial \eta }\right)$$

(32) $$\frac{\partial^{2}\phi_{m}}{\partial \eta^{2}}+\frac{1}{\eta }\frac{\partial \phi_{m}}{\partial \eta }=0$$

and the corresponding boundary conditions are

(33) $$\phi_{a}(\eta ,\;0)=1,\;0\leq \eta \leq ∆$$

(34) $$\frac{\partial \phi_{a}(0,\xi )}{\partial \eta_{a}}=0,\;0\leq \xi \leq 1$$

(35) $$\theta \phi_{a}(1,\xi )=\phi_{m}(0,\xi ),\;0\leq \xi \leq 1$$

(36) $$D\frac{\partial \phi_{a}(∆,\xi )}{\partial \eta }=D_{m}\frac{\partial \phi_{m}(∆,\xi )}{\partial \eta },\;0\leq \xi \leq 1$$

(37) $$\phi_{b}({∆}_{1},\xi )=\phi_{m}({∆}_{1},\xi ),\;0\leq \xi \leq 1$$

(38) $$D\frac{\partial \phi_{b}({∆}_{1},\xi )}{\partial \eta }=D_{m}\frac{\partial \phi_{m}({∆}_{1},\xi )}{\partial \eta },\;0\leq \xi \leq 1$$

(39) $$\frac{\partial \phi_{b}(1,\xi )}{\partial \eta }=0,\;0\leq \xi \leq 1$$

(40) $$\phi_{b}(\eta ,\;0)=\phi_{\mathrm{bI}},\;{∆}_{1}\leq \eta \leq 1$$

The solute in membrane phase is transported by diffusion only, as shown in Figure 3 and indicated in Equation (32), which is called the Euler equation and can be solved easily by incorporating t=ln⁡η to transform as follows:

(41) $$\frac{\partial^{2}\phi_{m}}{\partial t^{2}}=0$$

Integrating Equation (41) twice to obtain the general solution as

(42) $$\phi_{m}(\eta ,\xi )=a_{1}t+a_{2}\varphi_{m}(\eta ,\xi )=a_{1}\ln\eta +a_{2}$$

Thus, the solute concentration distribution $\phi_{m}\left(\eta ,\xi \right)$ in the membrane of Equation (42) and the derivative of $\phi_{m}\left(\eta ,\xi \right)$ were obtained with the use of the boundary conditions of Equations (35) and (37), as

(43) $$\phi_{m}(\eta ,\xi )=\frac{\left[\left(\ln\eta -\ln\Delta_{1})\theta \phi_{aw}(\xi )+(\ln\Delta -\ln\eta )\phi_{bw}(\xi \right)\right]}{\ln\Delta -\ln\Delta_{1}}$$

(44) $$\frac{\partial \phi_{m}(\eta ,\xi )}{\partial \eta }=\frac{1}{\eta }\left(\frac{\theta \phi_{aw}(\xi )-\phi_{bw}(\xi )}{\ln\Delta -\ln\Delta_{1}}\right)$$

By substituting Equation (44) into the boundary conditions of Equations (36) and (38), the derivatives of $\phi_{a}$ and $\phi_{b}$ were obtained in Equations (45) and (46), respectively:

(45) $$\frac{\partial \phi_{m}(\eta ,\xi )}{\partial \eta }=\frac{1}{\eta }\left(\frac{\theta \phi_{aw}(\xi )-\phi_{bw}(\xi )}{\ln\Delta -\ln\Delta_{1}}\right)$$

(46) $$\frac{\partial \phi_{m}(\eta ,\xi )}{\partial \eta }=\frac{1}{\eta }\left(\frac{\theta \phi_{aw}(\xi )-\phi_{bw}(\xi )}{\ln\Delta -\ln\Delta_{1}}\right)$$

Hence, the two-dimensional solute concentration distributions of a concentric tubular dialysis-and-ultrafiltration operation can be solved by the governing equations, Equations (30) and (31), with the use of the boundary conditions of Equations (33), (34), (39), (40), (45) and (46).

## 3. Numerical Solutions for Solving Concentration Distributions

The mass balances of Equations (11) and (12) and Equations (30) and (31) were made for membrane dialysis along the flowing direction with and without ultrafiltration operations, respectively. Thus, the solute concentrations in both the inner and annular streams were solved numerically using the Crank–Nicolson method. The availability of computing software facilitates the numerical solution for this problem, in which the node numbers in the η-direction were N and J for the retentate and dialysate phases, respectively, and the node numbers in the ξ-direction were P for both the retentate and dialysate phases, when the iterative procedures reached the convergence tolerance. Hence, dialysis efficiency and dialysis rate improvement were obtained. Comparisons were made for the solute of dialysis efficiency with and without ultrafiltration operations. The partial differential equations have been discretized and transformed with routines incorporating the finite difference algorithm, which are expressed in Appendix C.

## 4. Dialysis Rate, Dialysis Efficiency and Dialysis Rate Improvement

The dialysis rate of the dialysis system with and without the ultrafiltration operation is defined as

(47) $$M=V_{aI}C_{aI}-V_{ao}C_{ao}$$

where $C_{ao}$ is the average outlet concentration of the retentate phase and $V_{ao}$ is the net outlet flow rate of the retentate phase ($V_{ao}=V_{aI}-V_{w}$). The dialysis efficiency of the dialysis system with the ultrafiltration operation is defined as the ratio of the dialysis rate to the maximum solute concentration gradient at the entrance as follows:

(48) $$\chi =\frac{M}{V_{\mathrm{aI}}(C_{\mathrm{aI}}-C_{\mathrm{bI}})}=\frac{1-(1-V_{w}/V_{\mathrm{aI}})\phi_{\mathrm{ao}}}{1-\phi_{\mathrm{bI}}}$$

Furthermore, the dialysis rate improvement $I_{D}(\%)$ of the dialysis system with the ultrafiltration operation was defined as the percentage increase in the dialysis efficiency χ relative to the dialysis efficiency of the pure dialysis system without the ultrafiltration operation:

(49) $$I_{D}(\%)=\frac{\chi -\chi_{0}}{\chi_{0}}\times 100\%$$

where the $\chi_{0}$ is the dialysis efficiency of the pure dialysis system without the ultrafiltration operation.

## 5. Results and Discussions

### 5.1. The Numerical Solutions Validated by Convergence Tolerance

The velocity distributions of $u_{a\xi }$ and $u_{b\xi }$ in the concentric tubular dialysis-and-ultrafiltration operation with the first- and second-order perturbation methods were obtained via comparison of the convergence tolerance, as demonstrated in Table 1 and Table 2, respectively. The selected order of perturbation methods indicated in Table 1 and Table 2 are dominant in this system. It was observed that only the first-order perturbation and zero-order perturbation in the inner subchannel and annular subchannel, respectively, need to be considered due to the convergence tolerance.

The two-dimensional theoretical model of the concentric tubular dialyzer with and without ultrafiltration operations was solved numerically using the Crank–Nicolson method, for which the convergence tolerance of the numerical solutions is shown in Table 3. The node numbers in the η-direction are N and J for the retentate and dialysate phases, respectively, and the node numbers in the ξ-direction are P for both the retentate and dialysate phases. The calculated results shown that two step sizes in the η-direction (N and J) and ξ-direction (P) agree reasonably well in the concentric tubular dialysis-and-ultrafiltration operation.

### 5.2. Concentration Distributions

**Figure 4:**
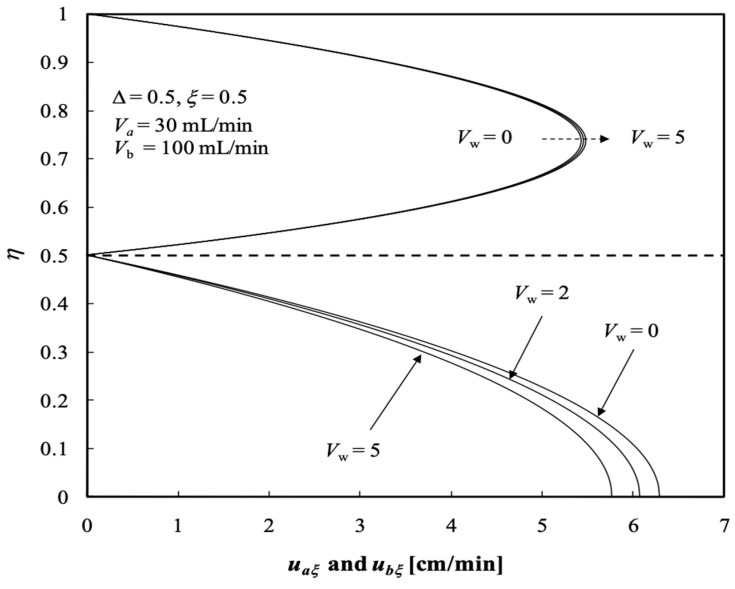
Axial velocity distributions in both retentate and dialysate phases.

**Figure 5:**
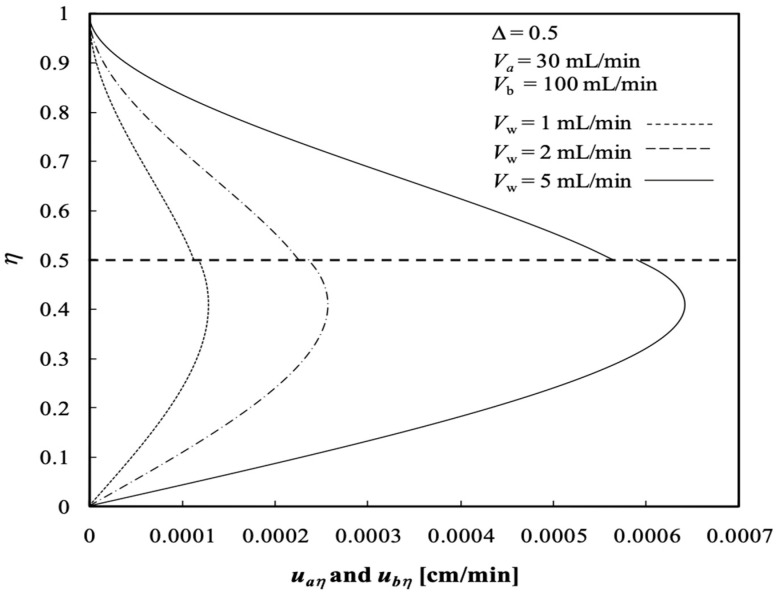
Radial velocity distributions in both retentate and dialysate phases.

The influences of the ultrafiltration rate $V_{w}$ on the axial velocities in the radial direction of both retentate and dialysate phases at the half axial position ξ=0.5 with ∆=0.5, are shown in Figure 4; both radial velocities vary with $V_{w}$ as a parameter, as indicated in Figure 5. The axial velocity in the retentate phase decreases with $V_{w}$ but the axial velocity in the dialysate phase increases with $V_{w}$. Meanwhile, both radial velocities in the retentate and dialysate phases increase with $V_{w}$. The findings shown in Figure 4 had previously been confirmed by Berman [33].

**Figure 6:**
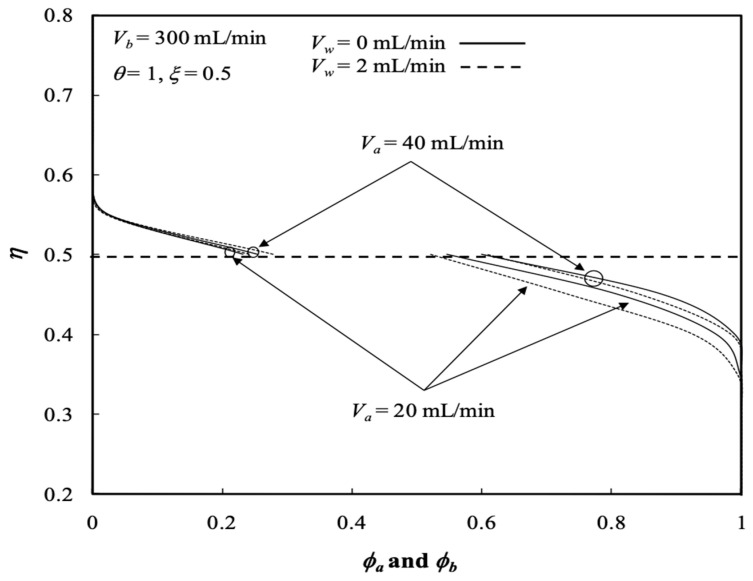
Concentration distributions of both retentate and dialysate phases with $V_{w}$ and $V_{a}$ as parameters.

**Figure 7:**
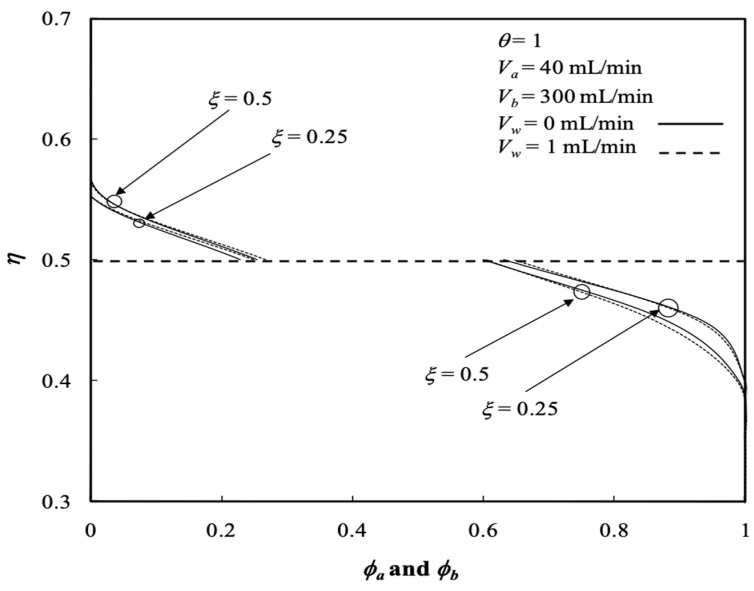
Concentration distributions of both phases with various longitudinal positions.

A smaller volumetric flow rate of retentate phase results in a lower concentration distribution due to the increase in the residence time for transporting the solute from the retentate phase to the dialysate phase, as shown in Figure 6. Comparisons were made regarding concentration distributions between the dialysis system with ($V_{w}$ = 1 mL/min) and without ($V_{w}=0\;mL/min$, as refers to the pure dialysis system without ultrafiltration) ultrafiltration rates in Figure 7, in which the results demonstrate the concentration distribution of the dialysis-and-ultrafiltration system decreases along the axial direction in the retentate phase, but increases in the dialysate phase.

**Figure 8:**
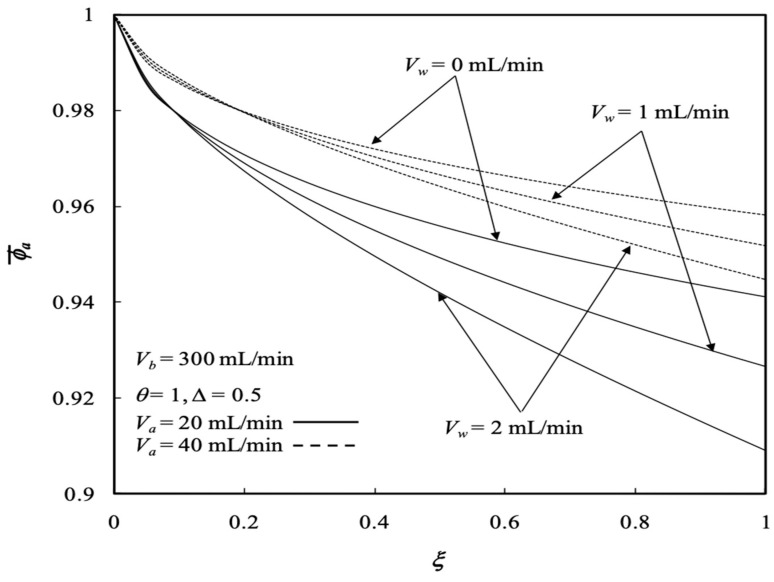
Average concentration distribution of the retentate phase with various ultrafiltration rates and volumetric flow rates of the dialysate phase.

**Figure 9:**
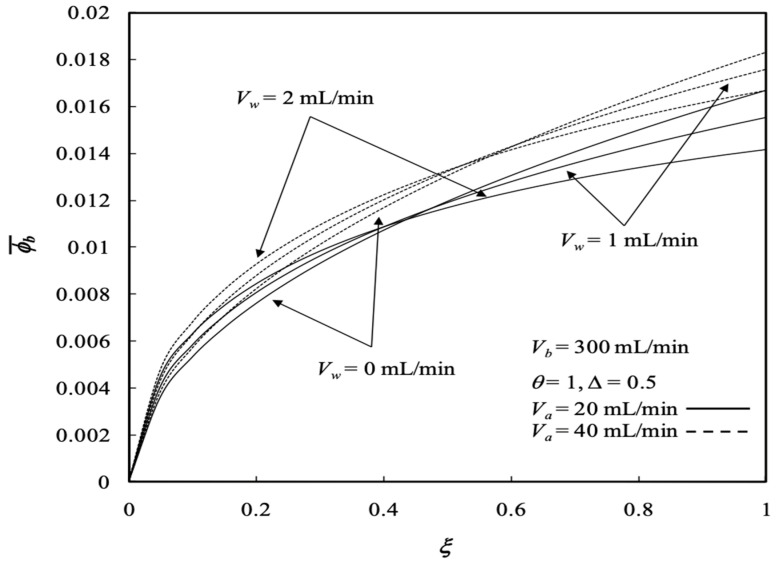
Average concentration distribution of the dialysate phase with various ultrafiltration rates and volumetric flow rates of the dialysate phase.

The influence of ultrafiltration rates and the retentate phase flow rates on the average concentrations of both the retentate and dialysate phases are presented in Figure 8 and Figure 9, respectively. The solute has a longer time to transport from the retentate phase to the dialysate phase with decreasing retentate phase flow rate due to increasing residence time under the lower retentate phase flow rate. Therefore, the average concentration of the retentate phase increases with increasing retentate phase flow rate but with decreasing ultrafiltration rate, as shown in Figure 8. The results indicate that the average concentration of the retentate phase may not decrease with increasing ultrafiltration operation owing to both the solute and solvent transporting through the membrane progressively and simultaneously. Average concentration distributions of the dialysis system in the retentate phase decrease along the axial direction and with the ultrafiltration rate, as shown in Figure 8. Figure 9 illustrates that the average concentration of the dialysate phase increases with increasing ultrafiltration rate and retentate phase flow rate. However, average concentration distributions in the dialysate phase increase along the axial direction as well as with the ultrafiltration rate except for the turning point by the inverse order with the ultrafiltration rate within 0.4 <ξ<0.6, as shown in Figure 9. Furthermore, average concentration distributions in both the retentate and dialysate phases increase with the volumetric flow rate of the dialysate phase accordingly.

**Figure 10:**
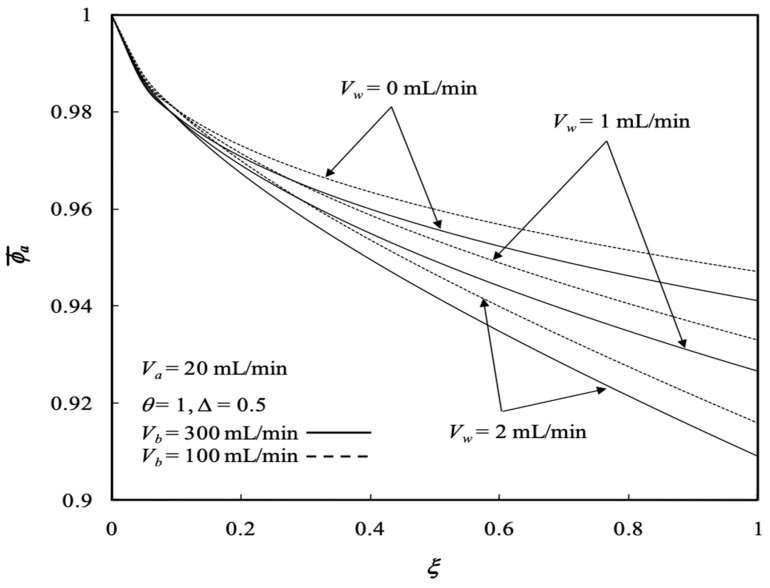
Average concentration distribution of the retentate phase with various ultrafiltration rates and volumetric flow rates of the retentate phase.

**Figure 11:**
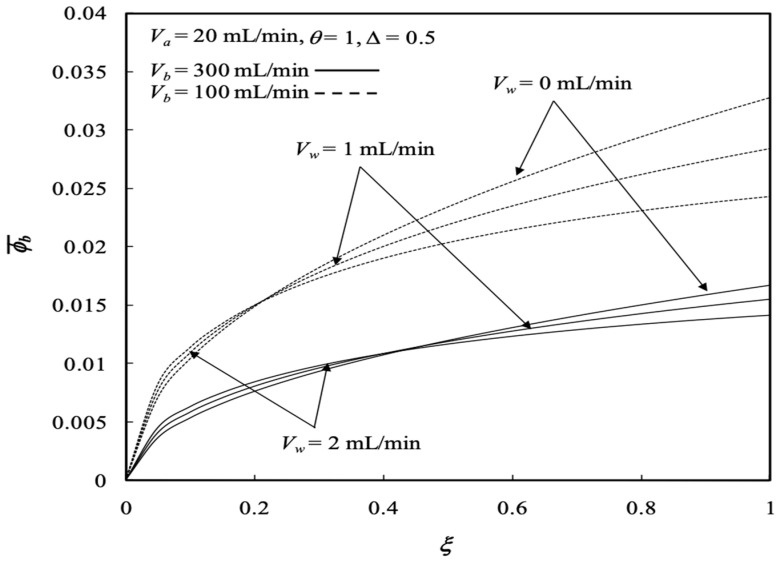
Average concentration distribution of the dialysate phase with various ultrafiltration rates and volumetric flow rates of the retentate phase.

**Figure 12:**
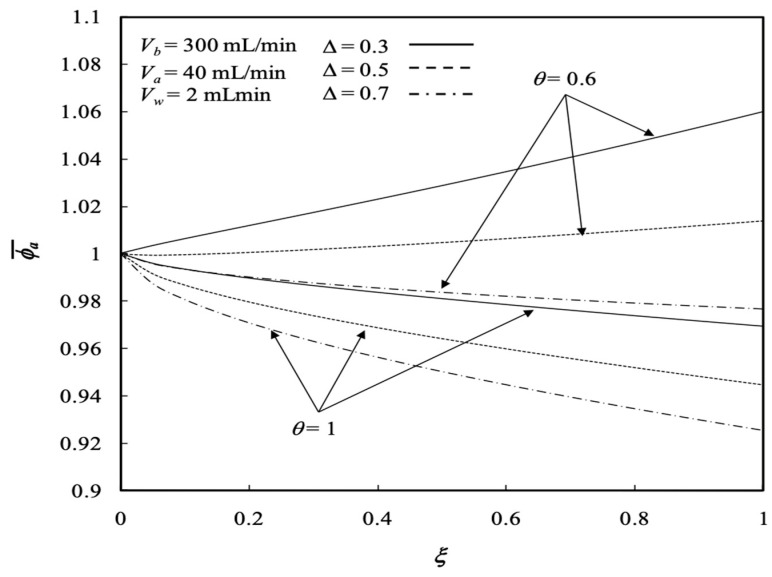
Average concentration distribution of the retentate phase with the membrane sieving coefficient and channel thickness ratio as parameters.

Similarly, the influences of various ultrafiltration rates and volumetric flow rates of the dialysate phase on both the retentate and dialysate phases are presented in Figure 10 and Figure 11, respectively. Average concentration distributions in the dialysate phase increase along the axial direction as well as with the ultrafiltration rate except for the turning point by the inverse order with the ultrafiltration rate shifting down to 0.2<ξ<0.4, as shown in Figure 12, and both average concentration distributions in the retentate and dialysate phases decrease with the volumetric flow rate of the dialysate phase. The effects of the membrane sieving coefficient θ and channel thickness ratio ∆ on the average concentration distribution of the retentate phase were examined with regard to the dialysis efficiency. Figure 12 shows the average concentration distribution of the retentate phase decreases with increases in both the membrane sieving coefficient and channel thickness ratio.

**Figure 13:**
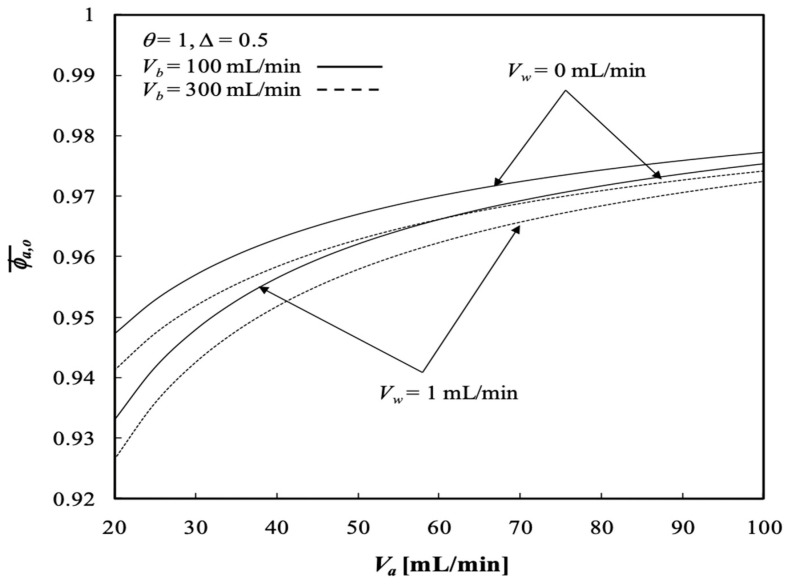
Average outlet concentration distribution of the retentate phase with the ultrafiltration rate and the retentate phase flow rate as parameters.

**Figure 14:**
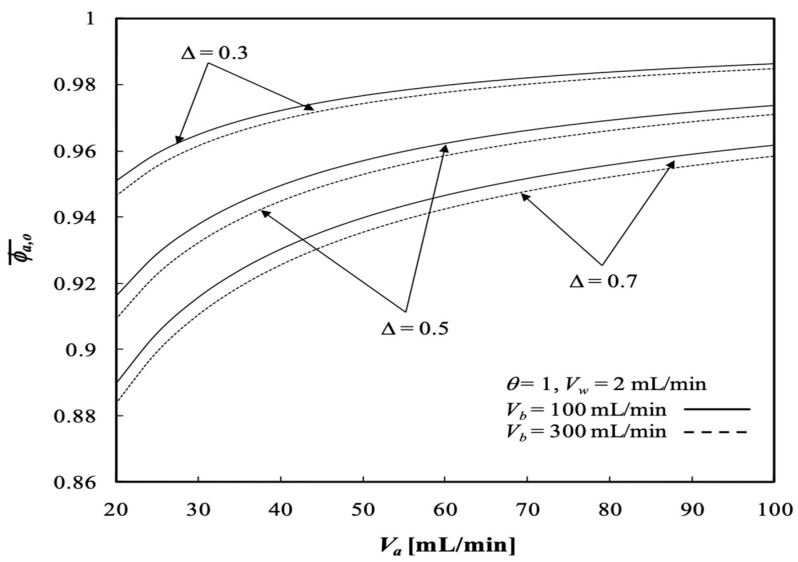
Average outlet concentration distribution of the retentate phase with the channel thickness ratio and the retentate phase flow rate as parameters.

The average outlet concentrations of the retentate phase $\bar{\phi_{a,o}}$ were studied with various ultrafiltration rates, channel thickness ratios and volumetric flow rates of the retentate phase as parameters, as indicated in Figure 13 and Figure 14. Figure 13 shows the average outlet concentration of retentate phase $\bar{\phi_{a,o}}$ increases with increasing retentate phase flow rate because the residence time is increased in the retentate phase. The results reveal that the average outlet concentrations of the retentate phase $\bar{\phi_{a,o}}$ decrease with increasing ultrafiltration rate and the volumetric flow rate of the dialysate phase, as indicated in Figure 13.

The effect of the channel thickness ratio ∆ on the average outlet concentration of the retentate phase $\bar{\phi_{a,o}}$ is demonstrated in Figure 14. The average outlet concentration of the retentate phase $\bar{\phi_{a,o}}$ decreases with decreasing channel thickness ratio, such as enlarging the annulus channel of the dialysate phase.

### 5.3. Dialysis Rate

**Figure 15:**
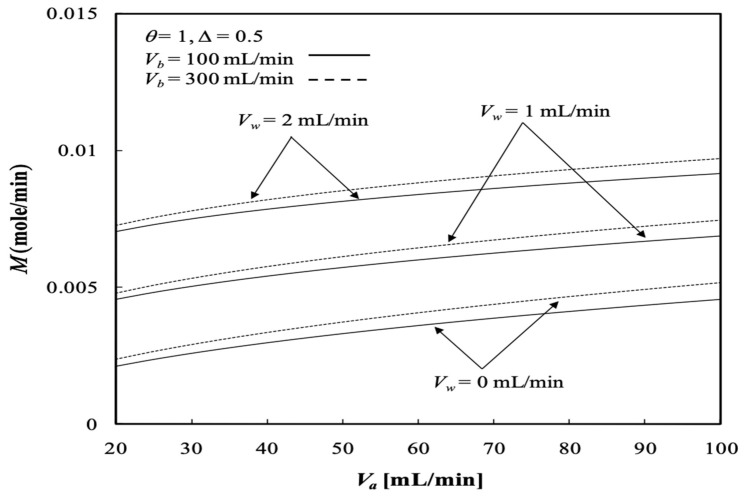
Dialysis rate with the ultrafiltration rate and the dialysate phase flow rate as parameters.

**Figure 16:**
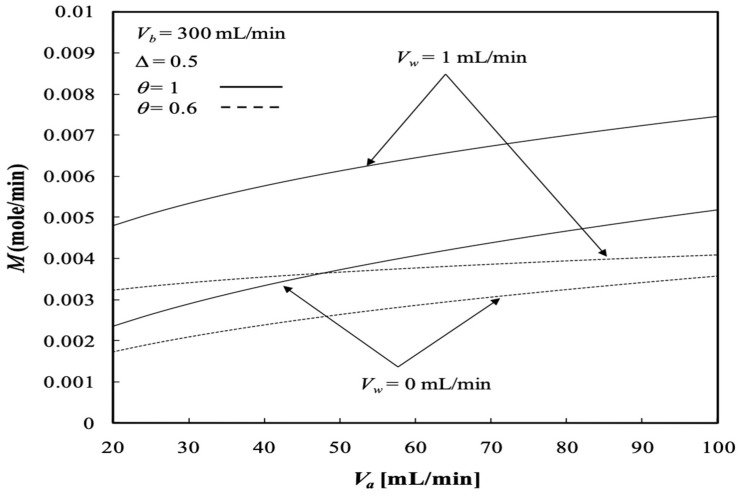
Dialysis rate with the membrane sieving coefficient and ultrafiltration rate as parameters.

The solute is transported through the membrane by two mechanisms in a dialysis module with ultrafiltration operation: (a) diffusion (caused by the concentration difference across the membrane) and (b) convection (caused by the ultrafiltration operation). The dialysis rate M of the concentric tubular dialysis-and-ultrafiltration system under ultrafiltration operation is calculated, as illustrated in Figure 15 and Figure 16.

The results show that the dialysis rate M increases with the retentate phase flow rate as well as the ultrafiltration rate in comparison to the system without ultrafiltration operation ($V_{w}=0$ when a pure dialysis system is employed). Meanwhile, Figure 15 indicates that the dialysis rate M increases with increasing retentate phase flow rate, which contributes to the average fluid velocity in the retentate phase and results in a higher convective mass transfer coefficient. This is attributed to the concentration gradient between the retentate and dialysate phases owing to the increasing dialysate phase flow rate, which thus results in an increased dialysis rate due to diffusion. Moreover, Figure 15 implies that the dialysis rate M increases with both the retentate and dialysate phase flow rates, which results in an increase in the convective mass-transfer coefficient in both the retentate and dialysate phases. The membrane sieving coefficient plays an important role in increasing the mass transfer rate by diffusing through the membrane, and thus, the dialysis rate M increases with the membrane sieving coefficient as well as the ultrafiltration rate and volumetric flow rate of the dialysate phase, as demonstrated in Figure 16.

**Figure 17:**
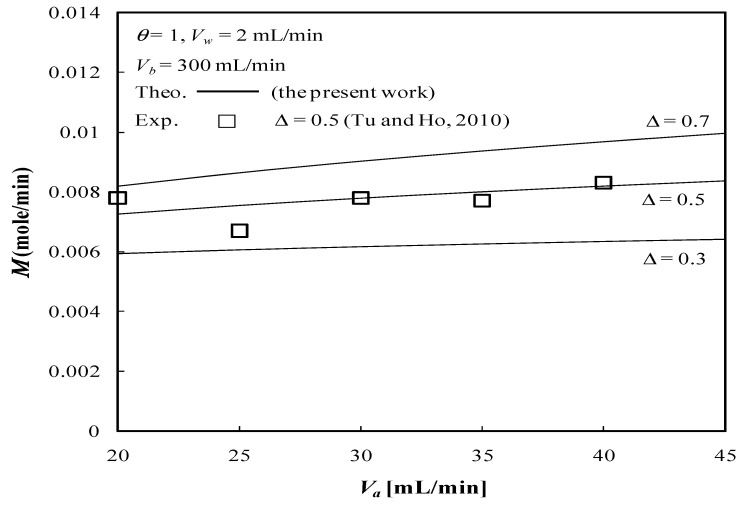
Comparisons of dialysis rate between theoretical results and experimental data [35] with the channel thickness ratio and the dialysate phase flow rate as parameters.

For the same reason as refers to the increase in the convective mass-transfer coefficient, the dialysis rate M also increases with increasing channel thickness ratio ∆, as shown in Figure 17.

The experimental work is cost prohibitive at the present time and there is only the available experimental data from the flat-plate dialyzer for confirmation. The present study could be analogized from those in our previous work on the flat-plate module [35]; this is a forthcoming work for our laboratory and will be conducted in the near future. In order to validate the theoretical predictions of the two-dimensional mathematical formulations in the concentric circular membrane dialyzer, a comparison was made with the experimental results conducted by the flat-plate dialyzer under ultrafiltration operation in our previous work [35] which neglected the tube curvature effect in the concentric tube [36]. The diminutive size, that is, ∆=0.7, of the annular spacing was relatively smaller than that of the tube diameters. Figure 17 denotes that the dialysis rate M also increases with the channel thickness ratio of both the concentric circular dialyzer (the present study) and flat-plate dialyzer [35] for comparisons. Restated, the geometry of this construction with negligible curvature effect can be approximated as that of a parallel-plate device with ∆=0.5. The theoretical predictions and experimental results of the dialyzer rate M are shown in Figure 17 within acceptable experimental deviations.

### 5.4. Dialysis Efficiency and Dialysis Rate Improvement

**Figure 18:**
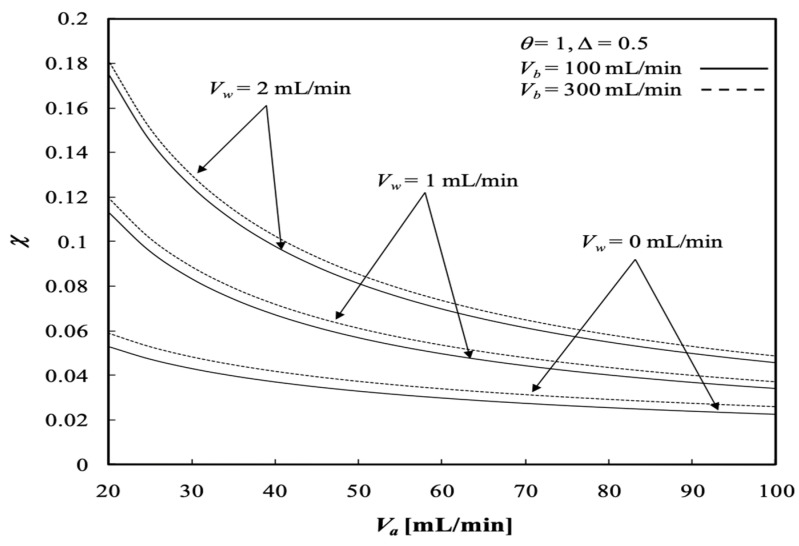
Dialysis efficiency with the ultrafiltration rate and the dialysate phase flow rate as parameters.

**Figure 19:**
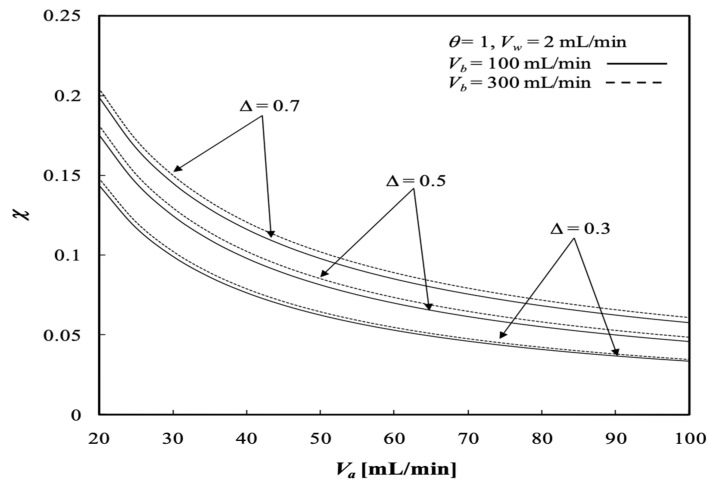
Dialysis efficiency with the channel thickness ratio and volumetric flow rate of the dialysate phase as parameters.

The dialysis efficiency χ of the concentric tubular dialysis-and-ultrafiltration system under ultrafiltration operation is expressed in terms of the ratio of the dialysis rate to the maximum solute concentration gradient at the entrance, as illustrated in Figure 18 and Figure 19, respectively.

The dialysis efficiency χ increases significantly with the ultrafiltration rate, especially under the lower volumetric flow rate of the dialysate phase, but decreases slightly with the volumetric flow rate of the retentate phase for the dialysis system under ultrafiltration operation, as shown by Figure 18. Results indicate that the ultrafiltration rate has a significant effect on the clearance of the solute, which has already been demonstrated by Jagannathan and Shettigar [22]. Figure 19 shows that the dialysis efficiency χ also increases with the channel thickness ratio. In addition, Figure 18 and Figure 19 indicate that the dialysis efficiency χ increases with the dialysate phase flow rate but decreases with increasing volumetric flow rate of the retentate phase.

**Figure 20:**
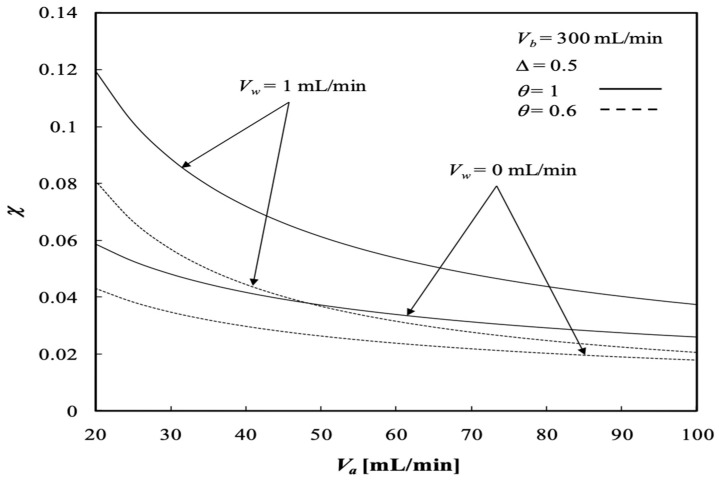
Dialysis efficiency with the membrane sieving coefficient and ultrafiltration rate as parameters.

The influence of the membrane sieving coefficient on the dialysis efficiency χ is presented in Figure 20. The results show that the dialysis efficiency χ increases with the membrane sieving coefficient and ultrafiltration rate but decreases with increasing volumetric flow rate of the retentate phase, as confirmed in Figure 20.

The dialysis rate improvements $I_{D}\left(\%\right)$ for θ=1 and ∆=0.5 were calculated as the relative percentage increment in concentric tubular dialysis-and-ultrafiltration module as compared with the module without ultrafiltration operation. The dialysis rate improvements $I_{D}(\%)$ are presented in Table 4 and Table 5, which show the results of the dialysis rate improvement of the concentric tubular dialysis-and-ultrafiltration module for various $V_{w}$ and ∆, respectively. The dialysis rate improvements $I_{D}(\%)$ increase with $V_{w}$ due to the increase in both diffusion and convective transfer through the membrane from the dialysate phase to the dialysate phase. The results also show that the dialysis rate improvements decrease with increasing ∆ as well as both $V_{a}$ and $V_{b}$. However, the maximum dialysis rate improved up to twice as compared with that of the pure dialysis system ($V_{w}=0)$ by employing a dialysis system with ultrafiltration operation, as shown in Table 4.

The dialysis rate improvements $I_{D}\left(\%\right)$ increase with ultrafiltration rate $V_{w}$ and membrane sieving coefficient θ but decrease with channel thickness ratio ∆ and both volumetric flow rates ($V_{a}$ and $V_{b})$ of the dialysate and retentate phases. The concentric tubular dialyzer with ultrafiltration operations transports more solute flux through the membrane of the tubular dialyzer with a higher dialysis rate. Generally, operating with the ultrafiltration rate shows a substantial influence which enhances the dialysis rate in the concentric tubular dialyzer. Several results can be concluded from Table 4 and Table 5, as follows. (1) The dialysis rate improvement $I_{D}\left(\%\right)$ increases with increasing ultrafiltration rate due to increasing convective mass transfer rate of the solute through the membrane; (2) the dialysis rate improvement $I_{D}\left(\%\right)$ decreases with increases in both the retentate phase flow rate and dialysate phase flow rate. The solute is transported through the membrane by diffusion and convection simultaneously in a dialysis-and-ultrafiltration operation, which means the contribution of the ultrafiltration rate on the dialysis rate is weakened when both the retentate phase and dialysate phase flow rates are increased.

## 6. Conclusions

A concentric tubular dialyzer with ultrafiltration operation to augment the dialysis rate was investigated theoretically. Two-dimensional mathematical equations were developed and formulated to predict the dialysis efficiency and dialysis rate improvement in the dialysis-and-ultrafiltration system as compared with the module without the ultrafiltration operation, which was solved numerically using the Crank–Nicolson method. There were two innovation points of this module design: (1) a two-dimensional mathematical model of the concentric tubular dialyzer was developed theoretically; (2) the simulated results show that the dialysis rate improvement was significantly improved with implementation of the ultrafiltration effect. A more direct method provides a straightforward strategy to the solution for determining the effect of the channel thickness ratio, which allows the specification setting by the designer to be met with economic consideration.

Regarding the pure dialysis system without ultrafiltration operation, its dialysis efficiency could be readily enhanced by employing the module with ultrafiltration operation, especially for operations with lower volumetric flow rate of the retentate phase. A maximum dialysis rate improvement of up to twice was found in the module with the ultrafiltration rate $V_{w}=2\;mL/min$ and membrane sieving coefficient θ=1 in comparisons with the module without ultrafiltration operation. The comparisons of the concentric tubular dialyzer with ultrafiltration operation relative to the module without ultrafiltration operations led to the following conclusions:

1. Average concentration distributions of the dialysis system in the retentate phase decrease along the axial direction and with decreasing ultrafiltration rate whereas the average concentration of the dialysate phase increases with increasing ultrafiltration rate and the retentate phase flow rate.
2. Both average concentration distributions in the retentate and dialysate phases decrease with the volumetric flow rate of the dialysate phase.
3. The average concentration distribution of the retentate phase decreases with increases in both the membrane sieving coefficient and channel thickness ratio.
4. The average outlet concentration of retentate phase increases with increasing retentate phase flow rate because the residence time is increased as well as with increasing ultrafiltration rate, channel thickness ratio and volumetric flow rate of the dialysate phase.
5. The results show that the dialysis rate M increases with increases in both retentate and dialysate phase flow rates, membrane sieving coefficient, ultrafiltration rate and channel thickness ratio relative to the system without ultrafiltration operation.
6. The dialysis efficiency χ increases with increasing dialysate phase flow rate, ultrafiltration rate, membrane sieving coefficient and channel thickness ratio but with decreasing volumetric flow rate of the retentate phase.
7. The dialysis rate improvements $I_{D}(\%)$ increase with increasing ultrafiltration rate and membrane sieving coefficient but with decreasing channel thickness ratio and both retentate and dialysate phase flow rates.

The results demonstrate the technical feasibility of dialysis rate improvement in the tubular membrane dialyzer with ultrafiltration operation. It was also found that the concentrations of the components (minerals and contaminants) in the retentate could be removed to reduce solutes in wastewater treatment processes, which depend on the separation technique and the operational parameters in membrane-based separation processes. In this paper, both the dialysis rate and ultrafiltration operation were examined from an economic perspective by implementing various ultrafiltration rates in the tubular dialyzer. Therefore, the alternative membrane sieving coefficient, membrane material and ultrafiltration rate require further investigation regarding the economic considerations of the tubular dialyzer. It is believed that the availability of such a simplified mathematical formulation as developed here is the value in the present work in designing a concentric tubular dialyzer and will be an important contribution to the design and investigation of multi-stream or multi-phase problems with coupling mutual boundary conditions. One may follow the present theory and develop a mathematical formulation to deal with multi-stream or multi-phase heat- or mass-transfer devices for each particular application with various geometries.

## Figures and Tables

**Table 1:** Comparisons of the first- and second-order perturbation methods for $u_{a\xi }$ (∆=0.5, $\xi =0.5,\;V_{a}=20\;mL/min$ ).

η	$V_{w}=1\;mL/min$		$V_{w}=2\;mL/min$	
	First Order	Second Order	First Order	Second Order
0.05	4.05	4.05	3.94	3.94
0.25	3.07	3.07	2.99	2.99
0.45	0.78	0.78	0.76	0.76

**Table 2:** Comparisons of the zero- and first-order perturbation methods for $u_{b\xi }$ (∆=0.5 , $\xi =0.5,V_{b}=100\;mL/min$ ).

η	$V_{w}=1\;mL/min$		$V_{w}=2\;mL/min$	
	Zero Order	First Order	Zero Order	First Order
0.55	1.16	1.16	1.17	1.17
0.75	5.46	5.46	5.48	5.48
0.95	1.82	1.82	1.83	1.83

**Table 3:** Comparisons of the convergence tolerance with the use of the Crank–Nicolson method (∆=0.5, $\theta =1,V_{w}=2\;mL/min$, $V_{a}=40\;mL/min,$ $V_{b}=300\;mL/min)$.

Dimensionless Concentration	*P* = 100		*P* = 200	
	*N* = *J* = 400	*N* = *J* = 500	*N* = *J* = 400	*N* = *J* = 500
$\phi_{aw}$	0.543	0.543	0.543	0.543
$\bar{\phi_{a,o}}$	0.945	0.945	0.945	0.945
$\phi_{bw}$	0.278	0.278	0.278	0.278
$\bar{\phi_{b,o}}$	0.017	0.017	0.017	0.017

**Table 4:** Dialysis rate improvement $I_{D}(\%)$ of the concentric tubular dialysis-and-ultrafiltration operation (∆=0.5 and $V_{b}=300\;mL/min)$.

$V_{a}$ (mL/min)	θ=1		θ=0.6	
	$V_{w}=1\;mL/min$	$V_{w}=2\;mL/min$	$V_{w}=1\;mL/min$	$V_{w}=2\;mL/min$
**20**	103.55	208.79	87.26	76.73
**30**	84.34	169.64	63.31	43.47
**40**	72.69	145.83	48.93	23.81

**Table 5:** Dialysis rate improvement $I_{D}(\%)$ of the concentric tubular dialysis-and-ultrafiltration operation ($V_{a}=40\;mL/min$ and $V_{w}=2\;mL/min)$.

∆	θ=1		θ=0.6	
	$V_{b}=100\;mL/min$	$V_{b}=200\;mL/min$	$V_{b}=100\;mL/min$	$V_{b}=200\;mL/min$
**0.3**	212.23	176.21	317.25	274.24
**0.5**	164.40	145.83	305.13	278.61
**0.7**	136.52	127.00	277.15	261.17

**Figure A1:**
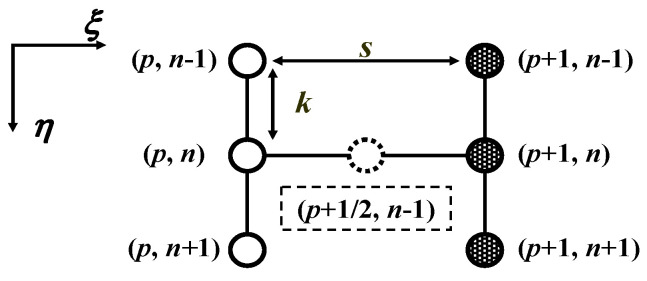
The scheme meshes of the Crank–Nicolson method. (s and k present the step sizes of ξ -direction and η -direction, respectively).

## Data Availability

Data are contained within the article.

## Abbreviations

C	Solute concentration in the stream (mol m^−3^)
D	Solute diffusivity in both retentate and dialysate phases (m^2^ s^−1^)
$D_{m}$	Solute diffusivity in membrane (m^2^ s^−1^)
$I_{D}$	Dialysis rate improvement
J	Node numbers in η-direction of dialysate phase
L	Length of membrane dialyzer (m)
M	Dialysis rate (mol s^−1^)
N	Node numbers in η-direction of retentate phase
P	Node numbers in ξ-direction
p	ξ-direction step sizes of the Crank–Nicolson method
$p_{a}$	Pressure in retentate phase (N m^−2^)
$p_{b}$	Pressure in dialysate phase (N m^−2^)
r	Transversal coordinate (m)
$r_{o}$	Outside radius of inner tube (m)
$r_{s}$	Inside radius of the shell tube (m)
$r_{t}$	Inside radius of inner tube (m)
Re	Reynolds number
$u_{a}$	Velocity distribution of the retentate phase (m s^−1^)
$u_{b}$	Velocity distribution of the dialysate phase (m s^−1^)
$\bar{u_{aI}}$	Averaged velocity of the retentate phase (m s^−1^)
$\bar{u_{bI}}$	Averaged velocity of the dialysate phase (m s^−1^)
$V_{aI}$	Inlet volumetric flow rate of the retentate phase (m^3^ s^−1^)
$V_{ao}$	Outlet volumetric flow rate of the retentate phase (m^3^ s^−1^)
$V_{bI}$	Inlet volumetric flow rate of the dialysate phase (m^3^ s^−1^)
$V_{bo}$	Outlet volumetric flow rate of the dialysate phase (m^3^ s^−1^)
$V_{w}$	Ultrafiltration rate (m^3^ s^−1^)
$u_{w}$	Velocity distribution of the dialysate phase (m s^−1^)
$v_{w}$	Ultrafiltration flux distribution (m s^−1^)
$v_{w0}$	Average ultrafiltration flux on the inner surface of the membrane (m s^−1^)
$v_{w1}$	Average ultrafiltration flux on the outer surface of the membrane (m s^−1^)
z	Axial coordinate along the flow direction (m)
*Greek letters*	
∆	Ratio of inside radius of inner tube to shell radius, $r_{t}/r_{s}$
${∆}_{1}$	Ratio of outside radius of inner tube to shell radius, $r_{o}/r_{s}$
δ	Membrane thickness (m)
ε	Membrane porosity
η	Dimensionless transversal coordinate
θ	Membrane sieving coefficient
$\lambda_{a}$	Wall Reynolds number of the retentate phase, $\lambda_{a}=\Delta r_{s}v_{w0}/\upsilon$
$\lambda_{b}$	Wall Reynolds number of the dialysate phase, $\lambda_{b}=\Delta_{1}r_{s}v_{w1}/\upsilon$
μ	Wall Reynolds number of the dialysate phase ($\mathrm{kg}\;{m^{-1}s}^{-1}$)
ξ	Dimensionless longitudinal coordinate
lρ	Density ($kgm^{-3}$)
lυ	Kinetic viscosity ($m^{2}s^{-1}$)
ϕ	Dimensionless solute concentration
$\overset{-}{\phi }$	Dimensionless average solute concentration
χ	Dialysis efficiency
φ	Stream function
*Subscripts*	
a	In the retentate phase
b	In the dialysate phase
I	At the inlet
o	At the outlet
w	On the membrane surface

## Appendix A

The corresponding boundary equations of Equations (3)–(6) are

(A1) $$\frac{\partial u_{az}(0,z)}{\partial r}=0,\;0\leq z\leq L$$

(A2) $$u_{az}(r_{t},z)=0,\;0\leq z\leq L$$

(A3) $$u_{az}(r,0)=2\bar{u_{aI}}(1-\frac{r^{2}}{r_{t}^{2}}),\;0\leq r\leq r_{t}$$

(A4) $$-p_{a}+\mu \frac{\partial u_{az}(r,1)}{\partial z}=0,\;0\leq r\leq r_{t}$$

(A5) $$u_{ar}(r_{t},z)=u_{br}(r_{o},z),\;0\leq z\leq L$$

(A6) $$u_{ar}(0,z)=0,\;0\leq z\leq L$$

(A7) $$u_{ar}(r,0)=0,\;0\leq r\leq r_{t}$$

(A8) $$\frac{\partial u_{ar}(r,1)}{\partial z}=0,\;0\leq r\leq r_{t}$$

(A9) $$u_{bz}(r_{o},z)=0,\;0\leq z\leq L$$

(A10) $$u_{bz}(r_{s},z)=0,\;0\leq z\leq L$$

(A11) $$u_{bz}(r,0)=2\bar{u_{bI}}\frac{\left[r_{s}^{2}-r^{2}+\left(r_{s}^{2}-r_{o}^{2}\right)\left(\frac{\ln r-\ln r_{s}}{\ln r_{s}-\ln r_{o}}\right)\right]}{\left[\frac{\left(r_{s}^{4}-r_{o}^{4}\right)}{\left(r_{s}^{2}-r_{o}^{2}\right)}-\frac{\left(r_{s}^{2}-r_{o}^{2}\right)}{\ln r_{s}-\ln r_{o}}\right]},\;r_{o}\leq r\leq r_{s}$$

(A12) $$-p_{b}+\mu \frac{\partial u_{bz}(r,\;1)}{\partial z}=0,\;r_{o}\leq r\leq r_{s}$$

(A13) $$u_{br}(r_{s},z)=0,\;0\leq z\leq L$$

(A14) $$u_{br}(r,0)=0,\;r_{o}\leq r\leq r_{s}$$

(A15) $$\frac{\partial u_{br}(r,1)}{\partial z}=0,\;r_{o}\leq r\leq r_{s}$$

in which $\bar{u_{aI}}=V_{aI}/\pi r_{t}^{2}$ and $\bar{u_{bI}}=V_{bI}/\pi (r_{s}^{2}-r_{o}^{2})$ are the average velocities for both inner subchannel *a* and annular subchannel *b*, respectively. Meanwhile, the average flow rate through the membrane was calculated as

(A16) $$v_{w}(r)=\frac{V_{w}}{2\pi rL},\;r_{t}\leq r\leq r_{o}$$

Then, the average ultrafiltration flux on the inner and outer surfaces of the membrane were evaluated accordingly as $v_{w0}=V_{w}/2\pi r_{t}L$ and $v_{w1}=V_{w}/2\pi r_{o}L$, respectively. Thus, Equation (A5) can be rewritten as follows:

(A17) $$u_{ar}(r_{t},z)=v_{w0}=\mathrm{constant},\;0\leq z\leq L$$

(A18) $$u_{br}(r_{o},z)=v_{w1}=\mathrm{constant},\;0\leq z\leq L$$

The stream function [25] of the inner subchannel was simplified by using the reservation of mass in the inner subchannel in terms of the dimensionless group $\eta^{*}={\left(r/r_{s}\right)}^{2}$ as

(A19) $$\varphi_{a}(\eta^{*},z)=[r_{t}^{2}\bar{u_{aI}}-2r_{t}v_{w0}z]f_{a}(\eta^{*})$$

The derivatives of Equation (A19) with respect to r and z, respectively, were obtained to give

(A20) $$\frac{\partial \varphi_{a}}{\partial r}=ru_{az}\;\mathrm{or}\;u_{az}=\left[2\Delta^{2}\bar{u_{aI}}-4\frac{\Delta v_{w0}}{r_{s}}z\right]f_{a}^{\prime }(\eta^{*})$$

(A21) $$\frac{\partial \varphi_{a}}{\partial z}=-ru_{ar}\;\mathrm{or}\;u_{ar}=\frac{2\Delta v_{w0}}{\sqrt{\eta^{*}}}f_{a}(\eta^{*})$$

in which $∆=r_{t}/r_{s}$. Substituting Equations (A20) and (A21) into Equations (3) and (4), Equations (3) and (4) can be rewritten as

(A22) $$\left(\frac{4}{r_{s}^{2}}\right)\left[2\Delta^{2}\bar{u_{aI}}-4\frac{\Delta v_{w0}}{r_{s}}z\right]\left\{\Delta v_{w0}r_{s}\left(f_{a}f_{a}^{\prime \prime }-(f_{a}^{\prime }{)}^{2}\right)-\upsilon \left(\eta^{*}f_{a}^{\prime \prime \prime }+f_{a}^{\prime \prime }\right)\right\}=-\frac{1}{\rho }\frac{\partial p_{a}}{\partial z}$$

and

(A23) $$\left(\frac{2\Delta v_{w0}}{r_{s}}\right)\left[\Delta v_{w0}r_{s}\left(\frac{2f_{a}f_{a}^{\prime }}{\eta^{*}}-\frac{f_{a}^{2}}{(\eta^{*}{)}^{2}}\right)-2\upsilon f_{a}^{\prime \prime }\right]=-\frac{1}{\rho }\frac{\partial p_{a}}{\partial \eta^{*}}$$

Combinations of the derivative of Equation (A22) with respect to r and the derivative of Equation (A23) with respect to *z* will obtain

(A24) $$\frac{\partial }{\partial \eta^{*}}\left\{\Delta v_{w0}r_{s}\left(f_{a}f_{a}^{\prime \prime }-(f_{a}^{\prime }{)}^{2}\right)-\upsilon \left(\eta^{*}f_{a}^{\prime \prime \prime }+f_{a}^{\prime \prime }\right)\right\}=0$$

or

(A25) $$\eta^{*}f_{a}^{\prime \prime \prime }+f_{a}^{\prime \prime }-\lambda_{a}\left[f_{a}f_{a}^{\prime \prime }-(f_{a}^{\prime }{)}^{2}\right]=k_{a}$$

in which $\lambda_{a}=\Delta r_{s}v_{w0}/\upsilon$ is the wall Reynolds number and $k_{a}$ is the integration constant. When the stream function of Equation (A19) was introduced into the boundary conditions, Equations (A1), (A2), (A6) and (A17) can be rewritten as

(A26) $$f_{a}^{\prime \prime }(0)=0$$

(A27) $$f_{a}^{\prime }(\Delta^{2})=0$$

(A28) $$f_{a}(0)=0$$

(A29) $$f_{a}(\Delta^{2})=1/2$$

The perturbation method [28] was used to solve the expressions of both $k_{a}$ and $f_{a}$ when $\left|\lambda_{a}\right|\leq 1$ as follows:

(A30) $$f_{a}=f_{a0}+\lambda_{a}f_{a1}+\lambda_{a}^{2}f_{a2}+...+\lambda_{a}^{n}f_{an}+...$$

(A31) $$k_{a}=S_{a0}+\lambda_{a}S_{a1}+\lambda_{a}^{2}S_{a2}+...+\lambda_{a}^{n}S_{an}+...$$

Applying the perturbation method to Equations (A30) and (A31) with the use of boundary conditions yields $f_{a}$ and the derivative ${f^{\prime }}_{a}$ once all constants ($f_{ai}$ and $k_{ai}$, i=0,1,2,…) were obtained as follows:

(A32) $$f_{a}=\frac{1}{\Delta^{4}}\left(\Delta^{2}\eta^{*}-\frac{1}{2}(\eta^{*}{)}^{2}\right)+\lambda_{a}\left[\frac{\eta^{*}}{18\Delta^{2}}-\frac{(\eta^{*}{)}^{2}}{8\Delta^{4}}+\frac{(\eta^{*}{)}^{3}}{12\Delta^{6}}-\frac{(\eta^{*}{)}^{4}}{72\Delta^{8}}\right]\;\;\;\;\;\;\;\;\;\;\;+\lambda_{a}^{2}\left[\frac{83\eta^{*}}{5400\Delta^{2}}-\frac{19(\eta^{*}{)}^{2}}{540\Delta^{4}}+\frac{11(\eta^{*}{)}^{3}}{432\Delta^{6}}-\frac{(\eta^{*}{)}^{4}}{144\Delta^{8}}\right.\left.+\frac{(\eta^{*}{)}^{5}}{720\Delta^{10}}-\frac{(\eta^{*}{)}^{6}}{10,800\Delta^{12}}\right]$$

(A33) $$f_{a}^{\prime }=\frac{\left(\Delta^{2}-\eta^{*}\right)}{\Delta^{4}}+\lambda_{a}\left[\frac{1}{18\Delta^{2}}-\frac{\eta^{*}}{4\Delta^{4}}+\frac{(\eta^{*}{)}^{2}}{4\Delta^{6}}-\frac{(\eta^{*}{)}^{3}}{18\Delta^{8}}\right]\;+\lambda_{a}^{2}\left[\frac{83}{5400\Delta^{2}}-\frac{19(\eta^{*})}{270\Delta^{4}}+\frac{11(\eta^{*}{)}^{2}}{144\Delta^{6}}-\frac{(\eta^{*}{)}^{3}}{36\Delta^{8}}\right.\left.+\frac{(\eta^{*}{)}^{4}}{144\Delta^{10}}-\frac{(\eta^{*}{)}^{5}}{1800\Delta^{12}}\right]$$

The dimensionless forms of velocity distributions, $u_{a\xi }$ and $u_{a\eta }$, of Equations (7) and (8) are obtained after substituting Equations (A32) and (A33) into Equations (A20) and (A21) accordingly.

## Appendix B

The stream function of the annulus subchannel was simplified by using the reservation of mass as

(A34) $$\varphi_{b}(\eta^{*},z)=[(r_{s}^{2}-r_{o}^{2})\cdot \bar{u_{bI}}+2r_{o}v_{w1}z]f_{b}(\eta^{*})$$

The derivatives of Equation (A34) with respect to r and z, respectively, were obtained to give

(A35) $$\frac{\partial \varphi_{b}}{\partial r}=ru_{bz}\;\mathrm{or}\;u_{bz}=\left[2{\left(1-\Delta_{1}\right)}^{2}\bar{u_{bI}}-4\frac{\Delta_{1}v_{w1}}{r_{s}}z\right]f_{b}^{\prime }(\eta^{*})$$

(A36) $$\frac{\partial \varphi_{b}}{\partial z}=-ru_{br}\;\mathrm{or}\;u_{br}=\frac{2\Delta_{1}v_{w1}}{\sqrt{\eta^{*}}}f_{b}(\eta^{*})$$

in which $f_{b}(\eta )=f_{b0}(\eta )+\lambda_{b}f_{b1}(\eta )$ and

(A37) $$f_{b0}\left(\eta \right)=S_{b0}\left[\frac{1}{2}+W_{1}+\left(\frac{\eta^{4}}{2}-\eta^{2}\right)+W_{1}\left(\eta^{2}\ln\eta^{2}-\eta^{2}\right)\right]$$

and

(A38) $$f_{b1}\left(\eta \right)=\frac{S_{b1}}{2}\eta^{4}+I_{0}\left(\eta^{2}ln\eta^{2}-\eta^{2}\right)+I_{1}\eta^{2}+I_{2}\;+{S_{b0}}^{2}[\frac{\eta^{8}}{72}-\frac{\eta^{6}}{72}\left(6+5W_{1}\right)]-\frac{{W_{1}\eta }^{2}}{2}\left(1+2W_{1}\right)\;+\frac{W_{1}}{12}\eta^{2}{ln\eta }^{2}(6-12\eta^{2}+\eta^{4}+12W_{1}-36W_{1}\eta^{2})\;\;\;\;\;\;\;\;\;\;\;\;\;\;\;\;+\frac{W_{1}}{4}\eta^{2}{{(ln\eta }^{2})}^{2}(-1-2W_{1}+2W_{1}\eta^{2})+\frac{W_{1}\eta^{2}}{4}(1+10W_{1}+24W_{1}^{2})$$

in which $I_{0}$, $I_{1}$ and $I_{2}$ are integration constants, and $S_{b0}$ and $S_{b1}$ are obtained as follows:

(A39) $$S_{b0}=\frac{1}{ln{∆}_{1}^{2}(1-{∆}_{1}^{4})+2{\left(1-{∆}_{1}^{2}\right)}^{2}}$$

(A40) $$\begin{array}{l} S_{b1}={-S}_{b0}^{2}\left[\frac{11}{36}+W_{1}+9W_{1}^{2}\right]\\ +\frac{S_{b0}^{2}}{W_{2}}\left[\frac{9W_{1}^{2}}{36}-\frac{4{∆}_{1}^{2}}{39}+\frac{11{∆}_{1}^{6}}{36}-\frac{{∆}_{1}^{8}}{8}+W_{1}(\frac{{∆}_{1}^{2}}{8}-\frac{{∆}_{1}^{4}}{4}+\frac{{∆}_{1}^{6}}{8})\right]\\ +\frac{{ln{∆}_{1}^{2}S}_{b0}^{2}}{W_{2}}\left[\frac{1}{36}+\frac{4{∆}_{1}^{4}}{72}-\frac{{∆}_{1}^{6}}{6}+\frac{{∆}_{1}^{8}}{24}-W_{1}\left(\frac{1}{144}+\frac{3{∆}_{1}^{2}}{2}+\frac{29{∆}_{1}^{4}}{16}+\frac{11{∆}_{1}^{6}}{36}\right)+W_{1}^{2}(\frac{1}{2}-4{∆}_{1}^{2}+\frac{7{∆}_{1}^{4}}{2})\right]\\ +\frac{{{\left(ln{∆}_{1}^{2}\right)}^{2}S}_{b0}^{2}}{W_{2}}\left[W_{1}\left(-\frac{1}{4}-\frac{{∆}_{1}^{2}}{4}-{∆}_{1}^{4}+\frac{{∆}_{1}^{6}}{6}\right)+W_{1}^{2}(-\frac{1}{2}+\frac{{∆}_{1}^{2}}{2}+(\frac{ln{∆}_{1}^{2}}{2}-3){∆}_{1}^{4}\right] \end{array}$$

where $W_{1}=\frac{1-{∆}_{1}^{2}}{ln{∆}_{1}^{2}}$ and $W_{2}=1-2{∆}_{1}^{2}+{∆}_{1}^{4}+\frac{ln{∆}_{1}^{2}}{2}(1-{∆}_{1}^{4})$

## Appendix C

The partial differential equations have been discretized and transformed with routines incorporating the finite difference algorithm, as shown in Figure A1, as follows:

(A41) $$\frac{\partial \phi }{\partial \xi }=\frac{\phi_{p+1}^{n}-\phi_{p}^{n}}{s}$$

(A42) $$\frac{\partial \phi }{\partial \eta }=\frac{1}{2}\left[\frac{\phi_{p+1}^{n+1}-\phi_{p+1}^{n-1}}{2k}+\frac{\phi_{p}^{n+1}-\phi_{p}^{n-1}}{2k}\right]$$

(A43) $$\frac{\partial^{2}\phi }{\partial \eta^{2}}=\frac{1}{2}\left[\frac{\phi_{p+1}^{n+1}-2\phi_{p+1}^{n}+\phi_{p+1}^{n-1}}{k^{2}}+\frac{\phi_{p}^{n+1}-2\phi_{p}^{n}+\phi_{p}^{n-1}}{k^{2}}\right]$$
